# A signature of renal stress resistance induced by short-term dietary restriction, fasting, and protein restriction

**DOI:** 10.1038/srep40901

**Published:** 2017-01-19

**Authors:** F. Jongbloed, T. C. Saat, M. Verweij, C. Payan-Gomez, J. H. J. Hoeijmakers, S. van den Engel, C. T. van Oostrom, G. Ambagtsheer, S. Imholz, J. L. A. Pennings, H. van Steeg, J. N. M. IJzermans, M. E. T. Dollé, R. W. F. de Bruin

**Affiliations:** 1 Department of Surgery, Laboratory for Experimental Transplantation and Intestinal Surgery (LETIS), Erasmus University Medical Center, Rotterdam, the Netherlands; 2 Centre for Health Protection, National Institute for Public Health and the Environment, Bilthoven, the Netherlands; 3 Department of Genetics, Erasmus University Medical Center, Rotterdam, the Netherlands; 4 Facultad de Ciencias Naturales y Matemáticas, Universidad del Rosario, Bogotá, Colombia; 5 Department of Toxicogenetics, Leiden University Medical Center, Leiden, the Netherlands

## Abstract

During kidney transplantation, ischemia-reperfusion injury (IRI) induces oxidative stress. Short-term preoperative 30% dietary restriction (DR) and 3-day fasting protect against renal IRI. We investigated the contribution of macronutrients to this protection on both phenotypical and transcriptional levels. Male C57BL/6 mice were fed control food ad libitum, underwent two weeks of 30%DR, 3-day fasting, or received a protein-, carbohydrate- or fat-free diet for various periods of time. After completion of each diet, renal gene expression was investigated using microarrays. After induction of renal IRI by clamping the renal pedicles, animals were monitored seven days postoperatively for signs of IRI. In addition to 3-day fasting and two weeks 30%DR, three days of a protein-free diet protected against renal IRI as well, whereas the other diets did not. Gene expression patterns significantly overlapped between all diets except the fat-free diet. Detailed meta-analysis showed involvement of nuclear receptor signaling via transcription factors, including FOXO3, HNF4A and HMGA1. In conclusion, three days of a protein-free diet is sufficient to induce protection against renal IRI similar to 3-day fasting and two weeks of 30%DR. The elucidated network of common protective pathways and transcription factors further improves our mechanistic insight into the increased stress resistance induced by short-term DR.

Dietary restriction (DR) is a reduction in food intake without malnutrition^1^. Long-term DR is known to extend lifespan, increase overall health and improve resistance to multiple stressors in a wide variety of organisms^1^,^2^,^3^,^4^,^5^. Although the effect of DR on human lifespan is unknown, studies demonstrate a favorable impact on metabolic parameters associated with long-term health^6^,^7^,^8^. In addition, DR has been shown to protect against acute stressors including toxic chemotherapy^9^, paracetamol intoxication^10^, and oxidative stress induced by ischemia-reperfusion injury (IRI)^1^,^11^,^12^. In clinical kidney transplantation, renal IRI is a major risk factor for organ damage which may result in acute kidney injury^13^, primary non-function^14^, delayed graft function^15^, and acute and chronic rejection^16^ of the graft. After kidney retrieval, cessation of the blood flow (ischemia) leads to hypoxia, nutrient deprivation, and accumulation of metabolic waste products^15^,^16^. Reperfusion of the ischemic kidney promotes the generation of reactive oxygen species, triggers apoptotic cell death, and promotes the activation of an inflammatory response resulting in profound tissue injury^17^. Prevention or amelioration of renal IRI could increase graft quality, and prolong graft survival. Unfortunately, no effective treatment to reduce or prevent IRI is currently available.

Using renal IRI as a model, we previously demonstrated that the benefits of DR on IRI are induced rapidly: two and four weeks of 30% preoperative DR as well as three days of fasting reduce renal injury and strongly improve survival and kidney function after renal IRI in mice^1^,^12^. Hence, DR is a potential intervention for living kidney donors to reduce IRI and improve the transplantation success rate. Whether the protective effect of short- and long-term DR is based on the reduction of calories per se, or specific nutrients, was first investigated in fruit flies, in which long-term protein restriction contributed more to lifespan extension than a reduction in carbohydrates^18^. In mice, glucose supplementation did not interfere with fasting-induced protection against renal IRI, which also points towards a role for specific (macro-)nutrients in inducing acute stress resistance^11^.

In this study, we investigated the role of specific macronutrients in inducing resistance against renal IRI by unrestricted feeding of protein-, carbohydrate-, and fat-free diets. We showed that the absence of protein for three days is sufficient to induce resistance against renal IRI and revealed common pathways and transcription factors that are implicated in the protective effect of calorie restriction, induced by two weeks of 30%DR, three days of fasting, and protein restriction.

## Results

### Absence of dietary protein induces protection against renal IRI

To determine the effect of short-term macronutrient deficiency on renal IRI, we provided 10-day, 14-day or 3-day regimens of protein-free, carbohydrate (CHO)-free and fat-free diets before inducing renal IRI.

As previously shown, mice that were fed a protein-free diet for six or 14 days tend to voluntarily decrease their food intake as compared to animals that were fed a normal diet^19^. We indeed found that mice fed a protein-free diet for 10 days decreased their dietary food intake by approximately 30% (Figure S1A), resulting in significant body weight loss up until 20% on day 10 (Figure S1B). The reduction in food intake and body weight was less substantial in mice fed a CHO-free diet for 14 days. A fat-free diet for 14 days led to a small increase in body weight. Only the protein-free diet improved survival (Figure S1C) and kidney function (Figure S1D) following renal IRI. However, due to the reduction in food intake and body weight, the effect of the absence of protein per se could not be disentangled from the effect of calorie restriction.

**Figure 1:**
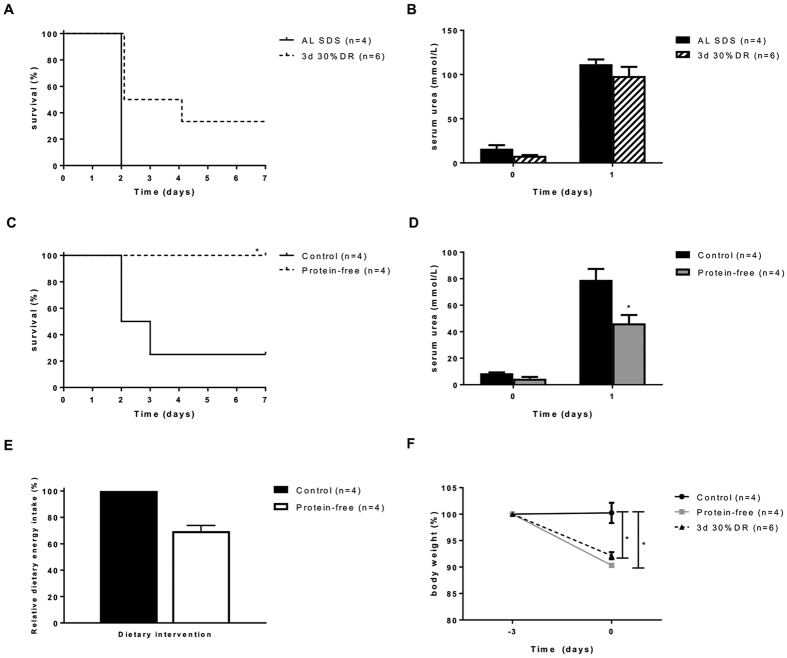
A protein-free diet protects against renal IRI. (**A**) Three days of 30%DR did not protect against renal IRI. (**B**) Serum urea levels before and one day after renal IRI did not differ between mice fed ad libitum (AL) for three days of 30%DR and a control diet. (**C**) Three days of protein-free diet improved survival compared with AL fed controls. (**D**) Serum urea levels of AL fed protein-free mice were significantly better compared to AL fed control mice (P < 0.05). (**E**) Three days of protein-free diet resulted in 30% calorie restriction. (**F**) Mice fed a protein-free diet or a 30%DR diet for three days lost significantly more body weight than mice fed the control diet. Body weight changes did not differ between the protein-free diet and 3-day 30%DR. AL Control =  ad libitum fed SDS chow, Control =  control diet for the macronutrient diets, protein free =  ad libitum fed protein-free diet. ^*^P < 0.05.

Subsequently, to separate the effect of the absence of protein from calorie restriction, mice were fed a protein-free diet for three consecutive days. We first showed that survival and kidney function of mice receiving 30%DR for three days did not differ from mice fed ad libitum (Fig. 1A,B)^11^. Mice fed the protein-free diet for three days had significantly improved survival (Fig. 1C) and kidney function compared to control mice (P < 0.05) (Fig. 1D). The energy intake during the 3-day protein-free dietary intervention was decreased, but did not significantly differ from the intake of animals fed the control diet for three days (P = 0.13) (Fig. 1E). Body weight of mice on the protein free diet decreased by 9%, while body weight of mice that were fed a control diet did not change. Mice receiving 30%DR for three days lost about 8% of their body weight (Fig. 1F).

### Common gene expression profiles between macronutrient-free diets, fasting and DR

To examine, in an unbiased manner, the transcriptomic response of the kidney, microarray analysis was performed on 45,141 probe sets in kidney samples after 3-days of fasting, three days and two weeks of 30%DR, three days of protein-, fat- or CHO-free diet, each compared to its corresponding AL fed control group. As the three days of fasting on the one hand and two weeks 30%DR and 3-day 30%DR on the other hand were each performed as individual cohorts using their own control group, we had three different control groups for all interventions combined. No significant differences in gene expression profiles were observed between the different control diets (data not shown). The highest number of significantly differentially expressed probe sets (DEPS) was found after three days of fasting, namely 2,604, of which 1,268 were up- and 1,336 down-regulated. Two weeks of 30%DR gave rise to a five times lower number: 492 DEPS, of which 265 were up-regulated and 227 were down-regulated. Three days of protein-free diet induced 391 DEPS, with 230 probe sets up- and 161 down-regulated. Of the non-protective diets, the 3-day fat-free diet did not induce any DEPS when compared to control diet fed mice, while three days of a CHO-free diet induced 1,717 DEPS containing 613 up- and 1,104 down-regulated sets. Three days of 30%DR resulted in 454 DEPS, of which 284 up- and 170 down-regulated.

**Figure 2:**
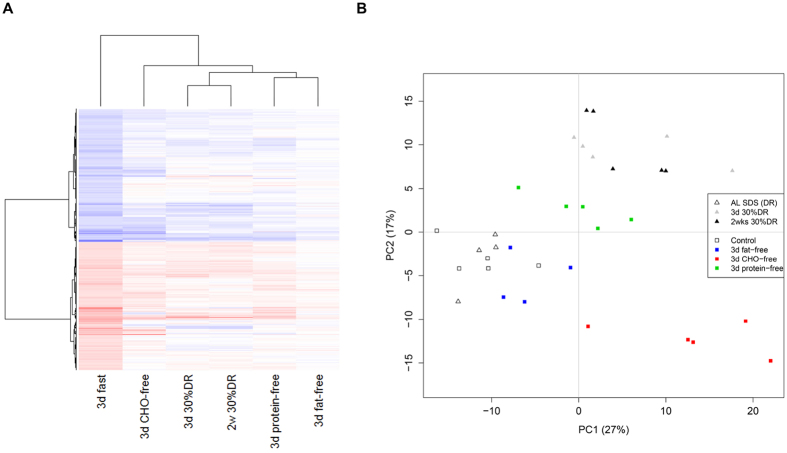
Heat map and PCA plots of directional and cluster patterns in all dietary interventions based on the differentially expressed probe sets (DEPS) after three days of fasting compared to its control group. (**A**) The majority of the DEPS in the kidney after three days of fasting showed the same directionality in the other dietary interventions. The 3-day CHO-free diet was the least clustered with the other diets, followed by two weeks 30%DR and a 3-day protein-free diet. The fat-free diet, showing no significant DEPS, clustered together with a 3-day protein-free diet. Red =  up-regulation, blue  =  down-regulation, white  =  no change. CHO-free  =  carbohydrate-free. (**B**) Principal component analysis (PCA) plot, based on the 2604 significantly regulated probe sets after three days fasting compared to the control diet fed animals. Both two weeks 30%DR and three days of 30%DR diet clustered close to each other based on the DEPS found after three days of fasting. The two control diets, control and SDS diet, clustered together with the non-protective fat-free diet. Three days of CHO-free diet positioned closely to two weeks and three days of 30%DR, but did not overlap with these diets. The protein-free diet had its own cluster, separated from the other groups on both PC axes.

Several analytic approaches were performed to compare the transcriptomic response between all diets. First, a comparison was made based on the number of overlapping DEPS and the corresponding significance of the overlap (Table 1). Based on the maximum relative overlap, the 3-day protein-free diet and 3-day fasting demonstrated the most resemblance to each other with 222 DEPS in common, corresponding to 53.3% relative overlap. Two-weeks 30%DR showed 45.9% relative overlap with three days of fasting, with 247 DEPS in common. To compare the significance of these overlapping fractions, the enrichment factors (EF) with corresponding p-values were calculated. Comparison of the two identically modified diets differing only in intervention time, namely 3-days 30%DR and two weeks 30%DR, resulted in the highest EF with a 39 times higher number of DEPS in common than would have been expected by chance (Table 1). Comparison of the 3-day protein-free diet with 3-day 30%DR resulted in an EF of 29. All enrichments were significant, with values lower than 8.66E-48, suggesting a significant common transcriptomic response between dietary interventions with the exception of a 3-day fat-free diet. As directionality of the DEPS was not accounted for in this comparison, we explored dendographic heat maps. Since the 3-day fasting dataset was hybridized on a different date, the date of hybridization caused a stronger effect than the biological signal (Figure S2). It was therefore not possible to integrate all complete datasets in one informative heat map analysis. As a solution, we assumed 3-days of fasting to represent the widest transcriptomic protective response, and all data sets were limited to its highest number of 2,604 DEPS. The resulting heat map (Fig. 2A) shows that the majority of the DEPS have similar directionalities, with the lowest expression levels in the fat-free diet. The horizontal dendogram, based on the (dis)similarity between expression data for probe sets, shows that three days of fasting portrays the largest differences with the other groups. However, this was an assumption, since the heat map is based on the probe sets differentially regulated after three days of fasting. Compared to 3-days of fasting, a 3-day CHO-free diet is the least clustered with the other dietary groups. The 3-day protein-free diet clusters closely with the non-protective 3-day fat-free diet, based on the number of probe sets as well as the expression levels. Three days 30%DR had a similar cluster pattern as two weeks of 30%DR.

Subsequently, the same set of 2,604 DEPS significantly regulated after fasting compared to AL fed controls, was used for a principal component analysis (PCA) among the macronutrient free diets, two weeks-, and three days 30%DR and their control diets (Fig. 2B). Samples from the control diets and the 3-day fat-free diet cluster together, with high overlap and corresponding directionality between the groups. With a large distance on the principal component (PC) 2 axis, a similar clustering was seen among the 3-day 30%DR and two weeks 30%DR samples. The protein-free diet had its own cluster, separated on both PC axes from the other groups. The 3-day CHO-free diet resembled the DR diets, but showed no overlap with these diets and showed a large dissimilarity to the other groups.

**Figure 3:**
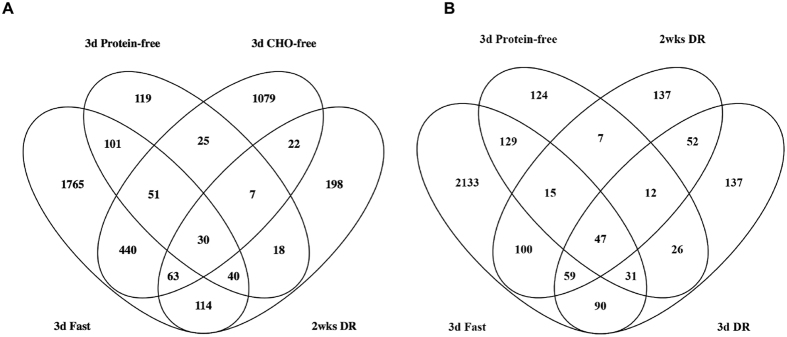
Venn diagram of multiple dietary interventions combined. **(A)** Venn diagram showing the number of DEPS after three days of fasting, two weeks 30%DR, three days of protein-free diet, three days of CHO-free diet, and their overlap with each diet. Thirty DEPS are differentially regulated in all four diets including the non-protective CHO-free diet (centre). The protective diets have 70 DEPS in common, of which 40 of those in common with the CHO-free diet are excluded (lower right). **(B)** Venn diagram showing the number of DEPS after three days of fasting, three days of a protein-free diet, two weeks and three days of 30%DR with their overlap. Forty-seven DEPS are differentially regulated in all fours diets (centre). A total of 15 DEPS were overlapping between the three protective diets.

To compare the transcriptomic responses between all dietary interventions, all DEPS were visualized in a Venn diagram (Fig. 3A). This revealed a total of 40 overlapping DEPS in the three protective diets, while 30 overlapping DEPS were also present in the non-protective CHO-free diet. The genes corresponding to the 70 DEPS in common are listed in Table S1. Comparing 3-day 30%DR with the protective diets, a similar pattern was observed; only 15 DEPS overlapped in the three protective diets, while 47 DEPS were also present in the non-protective 3-day 30%DR diet (Fig. 3B). However, both numbers of overlapping genes appeared too small to perform pathway analysis with the aim to find a common denominator of protection against renal IRI, and therefore an alternative approximation for analysis was used.

### Pathway analyses and transcription factor analyses

To explore the biological value of these transcriptomic responses, we used an individual approximation to identify significantly enriched pathways. The highest enriched pathways after the different dietary interventions were ranked by their –log p-value and summarized in Table S2. No clear pattern in overlapping pathways between all diets, or between the protective diets and the non-protective CHO-free diet, was observed. One pathway that emerged was the NRF2-mediated oxidative stress response pathway, since this pathway was activated by four out of five dietary interventions (Table S2).

To further identify a common protective response and dissect it from the response of the non-protective CHO-free diet, a more comprehensive meta-analytic approximation was used. A combining rank orders methodology was implemented to prevent bias of the results based on outliers as well as the stronger transcriptomic response after three days of fasting^20^. By eliminating all significant probe sets induced by the CHO-free diet in this meta-analysis, only 140 DEPS remained. Pathway analysis revealed no significant pathways (data not shown). Theorizing that the protective response might be partially overlapping with the response of the non-protective CHO-free diet, we repeated the meta-analysis with the three protective diets and the CHO-free diet into the approximation, thus not excluding the 3-day CHO-free diet. This meta-analysis yielded 640 DEPS. The majority of these DEPS in common showed a similar fold change and directionality among the three protective dietary interventions. A pathway analysis of these DEPS demonstrated regulation of nuclear receptor signaling as well as inhibition of cellular stress and injury and biosynthesis pathways amongst the top 10 overrepresented pathway categories (Table 2). Adding the 3-day 30%DR diet in the meta-analysis yielded 279 DEPS. No significant enriched pathways emerged. The 10 highest enriched pathways are depicted in Table S3.

To further explore the regulated DEPS and pathways in relation to the protective response against renal IRI, we examined the involvement of upstream transcription factors (TFs) in the DEPS that emerged from the meta-analysis. In the meta-analysis, 16 TFs were identified of which 12 were predicted to be activated and six inhibited (Table 3). Critical denominators might be TFs showing the same directionality in the protective diets, but are oppositely directed or not regulated in the non-protective CHO-free diet. The TFs complying with these criteria, in descending order of absolute z-score, were forkhead box O3 (FOXO3), heat shock factor protein 1 (HSF1), and high mobility group AT-hook 1 (HMGA1). Furthermore, hepatocyte nuclear factor 4-alpha (HNF4A) was highly activated in the protective diets, but only minimally in the non-protective CHO-free diet. Also, sterol regulatory element-binding transcription factor 1 (SREBF1) and 2 (SREBF2) were significantly down-regulated after 3-days of fasting and in the protein-free diet, activated in the CHO-free diet but not regulated after two weeks 30%DR. The non-protective 3-day 30%DR diet showed similar results as the protective diets, since all TFs after 3-days of 30%DR were similarly regulated as after three days of fasting, two weeks 30%DR and the 3-days of protein-free diet.

The validity of these findings was further examined by determining mRNA expression levels. The expression levels of Foxo1 were significantly higher after all diets except the fat-free diet (Figure S4A), while Foxo3 was significantly up-regulated in all diets except after two weeks of 30%DR (Figure S4B). Foxo4 was only significantly up-regulated after three days of protein-free and 3-days of CHO-free diet (Figure S4C). Hnf4α was not significantly regulated after any of the dietary interventions (Figure S4D). The mRNA expression level of down-regulated transcription factor Srebf1 was only significantly down-regulated after three days of fasting (Figure S4E), while Srebf2 was significantly down-regulated after three days of fasting and 3-days of protein-free diet (Figure S4F).

### Target pathways possibly involved in the protection against renal IRI

**Figure 4:**
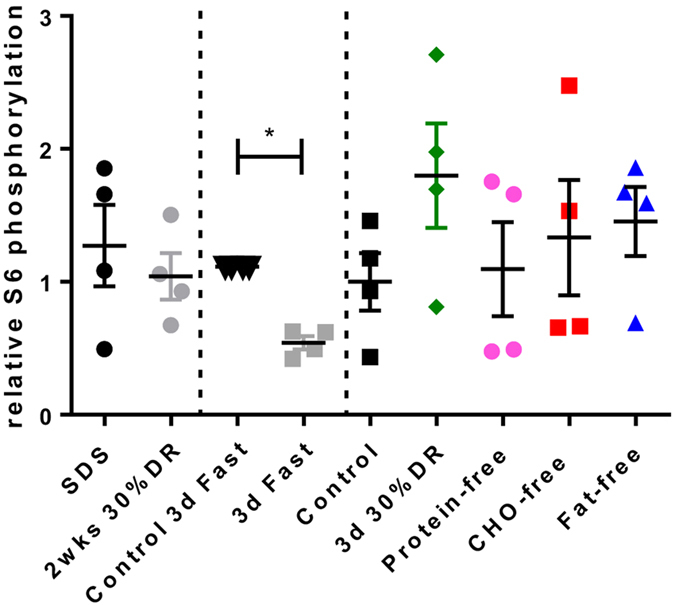
Relative S6 phosphorylation signals in kidney after various diet interventions. The ratio of phosphorylated S6 over total S6 was significantly lower after three days of fasting compared to its control diet. All other dietary interventions showed a large variation in phosphorylation levels that did not reach statistical significance.

Various pathways have been proposed to be involved in the protective response against renal IRI induced by DR and protein restriction. One of these is the eukaryotic translation factor 2 (eIF2α) signaling pathway, in which eIF2α is phosphorylated by the general control nonderepressible 2 (*Gcn2*) kinase, thereby inhibiting initiation of translation^19^. The role of *Gcn2* and the eIF2α pathway is subject of debate, and the relevance of this pathway still needs to be elucidated^19^,^21^. Our microarray analyses showed a significant upregulation of eIF2α transcription factor only after three days of fasting, while the Gcn2 gene and other target genes of the eIF2α pathway were not significantly differentially regulated after any of the dietary interventions. The mammalian Target of Rapamycin (mTOR) signaling pathway mediates between growth factors, hormones and nutrients to regulate essential cellular functions including survival and protein translation. Inhibition of the mTOR pathway has been demonstrated to increase lifespan in various animal species^22^,^23^ mTOR is part of mTOR complex 1 (mTORC1), a nutrient sensor complex that is involved in induction of oxidative stress resistance^24^. We found a downregulation of mTOR after two weeks 30%DR (−0.6; P < 0.01) and 3-days of protein-free diet (−1.7; P < 0.001) diets, while MTORC1 (−1.6; P < 0.01) and *mTOR* (−0.6; P < 0.001) were down-regulated after three days of fasting. Both non-protective diets 3-day CHO-free (+0.8; P < 0.01) and 3-day 30%DR (+1.3; P < 0.001) showed an up-regulation of mTOR. mTOR activity was examined at protein level by examining ribosomal protein S6 phosphorylation through immunoblotting in kidney extracts from all intervention and control groups. S6 is a down-stream target of mTOR through S6 kinase^25^. Compared to the corresponding control group, three days of fasting showed a significant two-fold increase in relative ribosomal protein S6 phosphorylation (Fig. 4). In the other dietary interventions, a large variation in phosphorylation levels was observed which did not reach statistical significance.

## Discussion

Since the discovery of the beneficial effects of short-term dietary restriction (DR) on stress resistance, optimizing its duration and content to eventually lead to a clinical applicable DR regimen has been an important part of the body of literature about DR^2^,^11^,^19^. Previously we have shown that two and four weeks of 30%DR as well as three days of fasting decreased morbidity and mortality, and improved kidney function in a murine renal IRI model^1^,^12^. In the present study, we show that a protein-free diet administered for only three days is sufficient to induce similar protection, whereas fat- and carbohydrate-free diets did not.

Initially, we attempted longer periods of diet interventions, but found that mice fed a protein-free diet during 10 days applied self-restriction of approximately 30% of their normal intake (Figure S1). Therefore, the possible beneficial effects of calorie and protein restriction on kidney function and survival were indistinguishable. In a recent publication, Peng *et al*. found that mice fed a protein-free diet for six days restricted calorie intake. However, they stated that corrected for body weight, their calorie intake was similar to that of ad libitum fed mice^19^. It is unknown whether six days of 30%DR induces protection against IRI, therefore a distinction between protein restriction and calorie restriction is still difficult to make. We showed that three days of 30%DR does not induce protection against renal IRI, and therefore we could disentangle the effects of calorie and protein restriction per se (Fig. 1). A protein-free diet, given for three days, induced protection whereas both a CHO-free and fat-free diet given for three or 14 days did not protect against renal IRI (Figure S1). A recent publication by Solon-Biet *et al*.^26^ showed that the ratio of proteins and carbohydrates rather than DR per se influenced the lifespan of mice as well as metabolic parameters such as insulin and lipids. Since proteins fully substituted the carbohydrates in our CHO-free diet, and carbohydrates fully substituted the proteins in our protein-free diet, these diets further emphasize the importance of the ratio between proteins and carbohydrates (Table S4).

Although the beneficial effects of both long-term and short-term DR have been acknowledged, the mechanisms underlying DR are still subject of investigation. Various pathways, factors and genes have been proposed to play a central role in the protective effects^27^, but attempts to validate these yielded conflicting results^28^,^29^,^30^,^31^. We produced extensive expression datasets of diets proven to be either protective or not protective against renal IRI. Gene expression profiles of the non-protective CHO-free diet showed a considerable overlap with gene expression profiles of protective diets, namely three days of fasting, two weeks 30%DR and 3-days of a protein-free diet (Fig. 3). A PCA plot demonstrated the partial different directionality of gene expression in the CHO-free diet compared to the other dietary interventions. The 3-day 30%DR diet also showed considerable overlap with the protective diets, mainly with two weeks 30%DR, and a partially different but also partially overlapping directionality. These findings could either indicate that overlapping probe sets and pathways are not involved in the induction of protection against renal IRI, or that they may require additional changes in other probe sets to induce this effect. Particularly, the striking similarity between three days and two weeks 30%DR suggests that three days is sufficient to activate a transcriptomic response which is not yet sufficient to induce phenotypical protection. In addition, the non-protective fat-free diet showed strong similarity with the protective 3-day protein-free diet, but with gene expression levels that were lower. These results indicate that the directionality, the number of probe sets and the expression levels are of importance in order to induce protection against renal IRI.

To provide structure in the infinite amount of data generated by microarray analysis, we used meta-analytic approximations to specify for overlapping pathways and factors in all diets. A specific meta-analysis in which the DEPS were oppositely regulated in the protective diets versus the non-protective CHO-free diet, resulted in only 160 DEPS and yielded no additional pathways of interest compared to a meta-analysis including the CHO-free diet. In this meta-analysis several TFs, including FOXO3 and HNF4A, remained significantly up-regulated in the protective diets whilst not or oppositely regulated in the CHO-free diet (data not shown).

Activation of nuclear receptor signaling pathways dominated the top 10 overrepresented pathways after three days of fasting, two weeks of 30%DR and 3-days of a protein-free diet (Table 2). Specific retinoid receptors, including the retinoid X receptor (RXR), the pregnane X receptor (PXR) and the peroxisome proliferator-activated receptor (PPAR)^32^, were also prominently up-regulated in our analysis, pointing towards a pivotal role for nuclear receptor signaling.

Nuclear receptors are transcription factors that can be activated by steroid hormones and lipid-soluble agents, such as the retinoid acids (RAs)^33^. Administration of RAs induces many of the beneficial effects observed after DR. DR is able to ameliorate age-related insulin resistance and degenerative brain diseases, and similar results have been described after treatment with RAs^34^,^35^,^36^. Both DR and administration of RAs are able to protect from ischemic stroke in the brain^37^,^38^,^39^. Signaling pathways activated by the interaction between RAs and nuclear receptors have also been considered to have tumor-, and immune suppressive effects, just as DR^9^,^33^,^40^.

Involvement of nuclear receptor signaling is further supported by the upstream TF analysis (Table 3), since the majority of the activated or inhibited TF could be directly or indirectly linked to the activation of nuclear receptors. The activated TFs in all protective diets that were oppositely or not regulated in the non-protective CHO-free diet are FOXO3, HNF4A, HMGA1 and HSF1. A role for each of these four TFs in increased stress resistance has been previously observed. For example, FOXO3 phosphorylation via the c-Jun N-terminal kinase -pathway results in its nuclear inclusion and activation of various processes involved in cellular stress resistance, biosynthesis, cell cycle regulation as well as apoptosis and autophagy^41^,^42^. A fasting-induced interaction, mediated by insulin signaling, with members of the FOXO family and RXR has been described as well^43^. HMGA1 is a downstream target of the insulin receptor pathway and could function as an important nuclear factor mediating the binding of FOXO proteins to other nuclear receptors, including the retinoid nuclear receptor family and thereby regulating insulin target genes^44^.

HNF4A is a nuclear TF that is involved in the development as well as in the metabolism of mainly the liver and kidney^45^. Up-regulation of HNF4A occurs via co-stimulation of LXR and FXR and usually depends on the presence of low levels of stressors, such as interleukin-1 and tumor necrosis factor alpha. Transcriptional activity of HNF-4a is also regulated indirectly by insulin through the action of FOXO1^45^. Activation of HNF4A results in inhibition of the activity of metabolic sensors, including SREBF and the mammalian target of Rapamycin (mTOR)^46^,^47^. This leads to down-regulation of metabolism, in particular cholesterol metabolism, and may contribute to increased stress resistance via the down-regulation of mTOR^47^,^48^. We showed that the activity of mTOR, as determined by the phosphorylation of the ribosomal protein S6, was significantly down-regulated after three days of fasting. A trend towards lower phosphorylation levels was seen after the other two protective diets: two weeks 30%DR and a 3-day protein-free diet. These data indicate that mTOR may play an important role in the protection against IRI, which may vary according to the type of nutrient deprivation that is offered.

Another pathway involved in nutrient sensing is GCN2, of which eIF2α is an important downstream target. GCN2 becomes transcriptionally activated by deprivation of amino acids and phosphorylates eIF2α, which leads to the activation of pathways involved in stress resistance. We did not find transcriptional regulation of the Gcn2 gene in any of the three protective diets. Although our data do not preclude posttranscriptional regulation of this pathway, they corroborate with those of Robertson *et al*.^49^, who observed that GCN2 signaling was not required for protection against renal IRI by protein restriction.

In summary, we demonstrated that three days of a protein-free diet in mice protects against renal IRI, similar to two weeks of 30%DR and three days of fasting. Comparative transcriptional analysis of kidney tissue following these dietary interventions demonstrated a significant overlap in differentially expressed genes and pathways, which are involved in resistance against oxidative damage induced by renal IRI. A meta-analysis of pathways and TFs indicates that DR up-regulates at least four TFs that activate a transcriptional response, which, in turn, increases nuclear receptor signaling dependent and independent cellular stress resistance. However, our attempt to understand the beneficial effects of different dietary restriction regimens on renal IRI by transcriptome analysis suggests that pivotal molecular changes also occur beyond the transcriptional level, and that additional “omics” analyses, including proteomics are needed to come to a complete understanding. Therefore, more research is warranted to further elucidate the role of these pathways in the induction of acute stress resistance by short-term DR, which may ultimately lead to “dietary restriction mimetic” therapeutic strategies that exploit the benefits of dietary restriction in humans.

## Materials and Methods

### Study design

Sample size calculation of our previous study was based on a 25% decrease of serum urea levels at time point 24 hours after renal IRI, with a standard deviation (SD) of 20% and a power of 0.8^1^,^11^. These experiments demonstrated that our renal IRI model was feasible and stable, and we therefore reduced the number of mice to six mice per group in the dietary intervention groups. Primary endpoint of this study was kidney function 24 hours after surgery, measured via serum urea concentrations. Secondary endpoints of this study were mortality rate in the first seven days after surgery and changes in gene expression profiles measured directly after each dietary intervention. Experimental data of two weeks 30%DR and three days of fasting groups were previously obtained^1^. Animals were euthanized and excluded from the experiment if their body weight decreased ≥20% of their preoperative weight or if they developed a moribund phenotype, including ruffled fur, hunched body posture, hypothermia, and decreased activity^11^,^12^.

### Animals

C57BL/6 male mice, 10–12 weeks old (20–25 grams), were obtained from Harlan, the Netherlands. Animals were housed in individually ventilated cages (3–4 animals/cage) under standard conditions. All mice had ad libitum (AL) food and water (acidified with HCl to a pH of 2.4–2.7) except where noted. All experiments were performed with the approval of the Animal Experiments Committee of the Erasmus University Medical Center, Rotterdam, the Netherlands under the Dutch National Experiments on Animals Act and according to the ARRIVE Guidelines, Animal Research: Reporting of *In Vivo* Experiments^50^.

### Diets and experimental design

The control chow for the fasting and dietary restriction groups was obtained from Special Diet Services (SDS, Witham, UK). All other diets were purchased from Research Diets, Inc. (New Brunswick, NJ, USA). The macronutrient composition and energy content of all diets are shown in Table S4. The control diet differed from the standard chow given (Special Diet Services, SDS) in the protein source, which consisted of lactic casein protein in case of control diet, while the SDS control consisted of crude protein. These two control diets are designated as “Control” and “SDS” throughout the manuscript.

#### Long-term dietary intervention

Upon arrival, mice were allowed *ad libitum* (AL) access to the SDS chow for seven days. At the start of the dietary intervention period, all mice were transferred to clean cages at 4:00 pm. Mice were randomly divided into a group with 30%DR (n = 5) or AL access to a carbohydrate-free (CHO-free) diet (n = 6) or a fat-free diet (n = 6) for 14 days, or a protein-free diet (n = 6) for 10 days (Figure S3). Mice in the control group for DR had AL access to the control SDS chow (n = 10), the control group for CHO-free and fat-free had AL access to the Control diet (n = 12). The effect of food intake was measured using pair-fed (PFed) control groups. Pair-feeding of each group was accomplished by giving the PFed groups the identical isocaloric amount of the control diet as the mice on the experimental diet had consumed the day before. The CHO-free, fat-free and protein-free diets were PFed in this manner (n = 6/group). Mice with 30%DR were given 70% of the normal daily intake of mice on the control diet, which was administered once daily at 4:00 pm. Since the phenotypical effects of two weeks 30%DR were reported previously, microarray analysis was used as the only outcome for this group^1^.

#### Short-term dietary intervention

Upon arrival, the same procedure was followed as for the long-term experiment. Mice were randomly divided into groups with AL access to control diet (n = 4), a protein-free diet (n = 6 per group) for three days or 30%DR for three days (n = 6), or into groups with AL access to SDS chow or fasting for three days (n = 5 per group). Since the phenotypical effects of three days fasting were reported previously, transcriptome analysis was the only parameter for this group^1^. For a graphical overview of the experimental setup see Figure S3.

### Dietary intake and body weight

Food intake and body weight were measured daily. To determine calorie intake, the daily food intake was corrected for the variation in the energy content (per gram of food) in the diets as follows: food intake per mouse times the number of calories per gram of food. Change in body weight was addressed in percentages calculated by dividing the body weight measurements during the dietary intervention through body weight at onset of the intervention period times 100.

### Surgical procedure

Following each dietary intervention, bilateral renal IRI was induced as previously described^1^. In brief, mice were anaesthetized via inhalation of isoflurane (5% isoflurane initially followed by 2–2.5% with oxygen for maintenance). Via midline abdominal incisions, the renal arteries and veins were exposed followed by occlusion of both renal pedicles for 37 minutes using non-traumatic vascular clamps. Purple discoloration of the kidneys confirmed ischemia macroscopically, while reperfusion was established when the color of both kidneys normalized after removal of the clamps. The incision was closed with 5/0 sutures in two layers. Following closure, mice received 0.5 ml PBS subcutaneously to compensation for fluid loss. The morning after surgery, another identical dose of PBS was given. Mice intended for microarray analysis were sacrificed immediately after the dietary intervention, without induction of renal IRI.

### Kidney function

Kidney function was determined as previously described by measuring serum urea levels in blood samples, collected via retro-orbital puncture while mice were anesthetized, before (T = 0) and day 1 (T = 1) after induction of renal IRI^1^.

### Immuno-blotting

Mouse kidney extracts from 9 different intervention diets were prepared by sonification with Soniprep 150 (Company, Place, Country) 2–3 times 30 seconds on ice in Laemmli buffer (135 mM Tris-HCl pH 6.8, 4.5% SDS, 22.5% glycerol), supplemented with complete protease inhibitors and PhosSTOP phosphates inhibitors (Roche Diagnostics, Indianapolis, IN, USA) After sonification, lysates were centrifuged at 4 degrees Celsius for 10 minutes. Protein concentrations were measured using the BCA Protein Assay Kit (Pierce Biotechnology, Rockford, USA). 25 μg protein was loaded on a NuPAGE 10% Bis-Tris Gel (Life Technologies LTD, Paisley, UK) and transferred to a PVDF Hybond-P (GE-Healthcare Life Sciences, Uppsala, Sweden) transfer membrane. Immunoblotting was performed with antibodies directed against S6 (#2217 S Lot5; 1:1.000) and Phospho-S6 (Ser240/244; #2215 Lot14; 1:500) (Cell Signaling Technology, Danvers, MA, USA). Blotting membranes were incubated with primary antibodies overnight at 4 degrees Celsius, before they were washed and incubated with 1:5000 diluted secondary anti-rabbit-IgG-HRP antibody (GE-Healthcare Life Sciences). Detection was performed by enhanced chemiluminescence using the ECL 2 Western Blotting Substrate (Pierce Biotechnology). Levels of S6 and Phospho-S6 were semi-quantified using the ImageJ software package (http://rsb.info.nih.gov/ij/index.html) and S6-phospho/S6-total ratios relative to the control diet were calculated and differences between groups were assessed with a t-test. Β-Actin was used as loading control (Sigma; A5441 Lot064M4789V; 1:25.000).

### Microarrays

The duration of dietary interventions that were used to study gene expression profiles was three days for all diets with exception of 30%DR, which was given for two weeks. Kidneys were obtained directly after each intervention and were snap frozen in liquid nitrogen until further analyses. An overview of the dietary interventions, the groups of mice and numbers used for phenotypical and transcriptional endpoints are summarized in Table S5. Total RNA was extracted using QIAzol lysis Reagent and miRNAeasy Mini Kits (QIAGEN, Hilden, Germany), following Qiagen protocol. Addition of wash buffers RPE and RWT (QIAGEN) was done mechanically by using the QIAcube (QIAGEN, Hilden, Germany) via the miRNAeasy program. Isolated RNA was and stored at −80 °C. The concentration of RNA was measured by Nanodrop (Thermo Fisher Scientific™, Breda, the Netherlands) and RNA quality was assessed using the 2100 Bio-Analyzer (Agilent Technologies, Amstelveen, the Netherlands) according to manufacturer’s instructions. The RNA quality was expressed as the RNA integrity number (RIN, range 0–10). RIN values of included samples ranged between 6.6 and 8.5. Hybridization to Affymetrix HT MG-430 PM Array Plates was performed at the Microarray Department of the University of Amsterdam (the Netherlands), according to Affymetrix protocols. Four to six biological replicates were used for each group. Quality control and normalization were performed using the pipeline at the www.arrayanalysis.org website (Maastricht University, the Netherlands)^26^. Normalization was done via the Robust Multichip Average (RMA) algorithm^51^. Due to a range in hybridization dates between fasting and the other diets (i.e. September 2011 versus August 2012), normalization of the data for fasted animals and their controls was done separately. Normalization output consisted of data for 45,141 probes, with several probes corresponding to the same Gene ID. Complete raw and normalized microarray data and their MIAME compliant metadata have been deposited at the Gene expression Omnibus (GEO) database GEO GSE65656 (www.ncbi.nlm.nih.gov/geo). After normalization, outliers were found in the control SDS (control 30%DR), the 3-day fasting and 3-day fat-free diet groups, defined as a deviation in array-array intensity correlations, principal component analysis and cluster dendograms. These outliers were excluded from further analyses (Table S5).

### Statistical analysis

Data are expressed as means ± standard error of the mean (SEM). Statistical analyses were performed using SPSS (version 21.0) and GraphPad Prism (version 5.0). Differences in serum urea concentrations were compared by Mann-Whitney U tests, food intake via the paired t-test and survival rates were analyzed by Log-rank tests. A p-value of  < 0.05 was considered significant. Microarray analyses were performed using the free software package R (R foundation). Gene expression data were compared using the Linear Models for Microarray Data (limma) method with correction for multiple testing using the false discovery rate (FDR) according to Benjamini and Hochberg^52^. Fold changes were expressed as the geometric mean per diet group against the corresponding ad libitum fed control group. Cutoff values for a significant difference were put at FDR  < 5% with fold change ≥1.5. The enrichment factor (EF) was calculated via the formula: EF = nAB/((nA × nB)/nC), where nA is the number of differentially expressed probe sets (DEPS) in experimental group A, nB the number of DEPS in experimental group B, nC the number of total genes in the microarray, and nAB the number of common DEPS between nA and nB. Data integration of microarrays hybridized in different dates was performed with meta-analysis. The methodology applied was combining rank orders. It is a non-parametric approach based on rank orders. The R package RankProd implemented in INMEX was used^20^. In summary, for each dataset, the fold changes (FC) were calculated for all possible pairwise comparisons. The ranks of the fold changes within each comparison were used to calculate the rank product for each gene. To assess the null distribution of the rank product within each data set, a permutation test was performed. The process was repeated several times to compute the P-value and false discovery rate (FDR) associated with each gene. A gene was selected as differentially expressed if it had an FDR  < 5% and an absolute combined FC ≥1.5. Functional annotation and analyses were performed using Ingenuity software (http://www.ingenuity.com/products/ipa). The prediction inhibition or activation of the upstream transcription regulators is calculated via de statistical z-score based on the observed gene expression changes in our dataset. Calculating the z-score reduces the chance of significant predictions based on random data (http://ingenuity.force.com/ipa/articles/Feature_Description/Upstream-Regulator-Analysis). Cutoff values for a significant activation or inhibition were met with a z-score of ≥2 or ≤−2, respectively.

## Additional Information

**How to cite this article:** Jongbloed, F. *et al*. A signature of renal stress resistance induced by short-term dietary restriction, fasting, and protein restriction. *Sci. Rep.* **7**, 40901; doi: 10.1038/srep40901 (2017).

**Publisher's note:** Springer Nature remains neutral with regard to jurisdictional claims in published maps and institutional affiliations.

## Supplementary Material

Supplementary Data

## Figures and Tables

**Table 1:** Comparison of the overlapping DEPS between the five dietary interventions and their corresponding P-value and enrichment factor.

Comparison	Overlapping DEPS	Relative overlap (%)*	Enrichment factor	*P*-value
Protein-free vs. Fasting	222	53.3	9.8	5.27E-169
2wks 30%DR vs. Fasting	247	45.9	8.7	8.88E-171
3d 30%DR vs. Fasting	208	42.0	8.7	3.12E-156
2wks 30%DR vs. 3d 30%DR	195	28.1	39.4	3.44E-270
Protein-free vs. CHO-free	113	24.9	6.0	5.84E-55
Fasting vs. CHO-free	584	23.7	5.9	3.73E-300
2wks 30%DR vs. CHO-free	122	20.4	8.2	5.71E-76
3d 30%DR vs. CHO-free	107	19.6	5.8	8.66E-48
Protein-free vs. 3d 30%DR	116	18.5	29.5	2.22E-138
Protein-free vs. 2wks 30%DR	95	15.2	22.3	1.15E-99

The highest percentage of relative overlap was found between three days of protein-free diet and two weeks of 30%DR. The enrichment factor indicates the number of times the overlapping DEPS is higher than expected by chance. All diets showed a significantly overlapping number of DEPS, as shown by the corresponding p-values in the last column. *Relative overlap is calculated by the number of DEPS in common between the two groups, divided by the total number of unique DEPS across both groups, relative to the theoretical maximum overlap according to this formula.

**Table 2:** Overview of the top 10 overrepresented canonical pathways in the meta-analysis ranked by their −log *P*-value.

*Meta-analysis*	Pathway Category	P-value	Genes Ratio	Z-score
Canonical Pathway				
LXR/RXR Activation	Nuclear Receptor Signaling	7.24E-09	16/121 (13.2%)	+0.302
FXR/RXR Activation	Nuclear Receptor Signaling	3.63E-06	13/127 (10.2%)	N/A
LPS/IL-1 Mediated Inhibition of RXR Function	Nuclear Receptor Signaling	2.24E-05	16/219 (7.3%)	−2.646
Superpathway of Cholesterol Biosynthesis	Fatty Acids and Lipids Biosynthesis, Sterol Biosynthesis	2.40E-05	6/28 (21.4%)	N/A
NRF2-mediated Oxidative Stress Response	Cellular Stress and Injury	3.72E-05	14/180 (7.8%)	−1.000
Aryl Hydrocarbon Receptor Signaling	Cell Cycle Regulation; Apoptosis; Xenobiotic Metabolism, Nuclear Receptor Signaling	5.13E-05	12/140 (8.6%)	+1.633
PXR/RXR Activation	Nuclear Receptor Signaling	9.12E-05	8/67 (11.9%)	N/A
Intrinsic Prothrombin Activation Pathway	Cardiovascular Signaling; Cellular Stress and Injury	3.47E-03	5/29 (17.2%)	N/A
Superpathway of Geranylgeranyldiphosphate Biosynthesis I	Cofactors, Prosthetic Groups and Electron Carriers Biosynthesis	3.98E-03	4/17 (23.5%)	N/A
Aldosterone Signaling in Epithelial Cells	Cardiovascular Signaling; Nuclear Receptor Signaling	4.57E-03	11/152 (7.2%)	N/A

The top 10 overrepresented pathways derived from the 640 DEPS in common between 3-days of fasting, two weeks 30%DR, three days of a protein-free diet and three days of a carbohydrate-free diet. These pathways are mostly involved in regulation of nuclear receptor signalling (five out of 10), biosynthesis signalling (two out of 10) and cellular stress and injury (two out of 10).

**Table 3:** List of upstream transcription factors after meta-analysis with corresponding z-scores in the different dietary interventions.

Dietary intervention	Meta-analysis	3-days fasting	2 weeks 30%DR	Protein-free	CHO-free	3 days 30%DR
Transcription Factor						
SMAD7 – SMAD family member 7	**+2.890**	+0.718	-0.128	**+2.466**	N/A	N/A
*FOXO3 - Forkhead box O3	**+2.729**	**+3.303**	+1.042	+1.701	−0.314	+1.893
FOXO1 - Forkhead box O1	**+2.692**	+1.599	−0.109	+0.269	−1.850	+0.360
*HNF4A – Hepatocyte nuclear factor 4 alpha	**+2.462**	**+2.122**	+1.179	**+2.089**	+0.109	+1.327
MYCN – v-myc avian myelocytomatosis viral oncogene	**+2.414**	+1.982	N/A	+1.245	**−2.464**	+1.091
CLOCK – Circadian Locomotor output Cycles Kaput	**+2.373**	+1.980	+1.119	N/A	+1.940	+1.925
*HMGA1 – High mobility group AT-hook 1	**+2.051**	+1.432	**+2.177**	+0.356	N/A	+0.785
MED1 – Mediator complex subunit 1	**+2.008**	**+2.058**	N/A	N/A	+0.102	N/A
SPDEF – SAM pointed domain containing ETS transcription factor	**+2.000**	**+2.170**	N/A	+1.000	N/A	N/A
LYL1 – Lymphoblastic leukemia associated hematopoiesis regulator 1	**+2.000**	**+2.000**	N/A	N/A	N/A	+1.982
SIM1 – Single-minded homolog 1	**+2.000**	+0.557	N/A	N/A	N/A	N/A
ARNT2 – Aryl hydrocarbon receptor nuclear translocator 2	**+2.000**	+0.557	N/A	N/A	N/A	N/A
GLI1 – GLI family zinc finger 1	**−2.183**	N/A	N/A	+0.057	N/A	N/A
*HSF1 – Heat shock factor protein 1	**−2.697**	−1.659	**−2.653**	**−2.376**	+0.462	−0.451
SREBF2 - Sterol regulatory element-binding transcription factor 2	**−2.923**	**−3.420**	N/A	−0.243	+0.660	N/A
SREBF1 - Sterol regulatory element-binding transcription factor 1	**−3.889**	**−2.794**	N/A	−1.304	+1.229	−0.779

Upstream regulator analysis of the DEPS found in common after 3-days of fasting, two weeks 30%DR and 3-days of a protein-free diet (meta-analysis) revealed 16 transcription factors (TFs) significantly regulated, of which 12 were activated and four were inhibited. Highest activated TFs were SMAD7 and FOXO3. *TFs of interest, that are not or oppositely regulated in the non-protective CHO-free diet. DR = dietary restriction; CHO = carbohydrate.
