# Supplementary material for: A signature of renal stress resistance induced by short-term dietary restriction, fasting, and protein restriction
Source: Sci Rep. 2017 Jan 19;7:40901. doi: 10.1038/srep40901 (PMC5244361; doi:10.1038/srep40901)
Supplement: Supplementary Data [file srep40901-s1.doc]

SUPPLEMENTARY DATA

A signature of renal stress resistance induced by short-term dietary restriction, fasting, and protein restriction

F. Jongbloed^1,2§^, T.C. Saat^1§^, M. Verweij^1^, C. Payan-Gomez^3,4^, J.H.J. Hoeijmakers^3^, S. van den Engel^1^, C.T. van Oostrom^2^, G. Ambagtsheer^1^, S. Imholz^2^, J.L.A. Pennings^2^, H. van Steeg^2,5^, J.N.M. IJzermans^1^, M.E.T. Dollé^2^, R.W.F. de Bruin^1*^

^§^ Authors contributed equally

CONTENTS:

- **Fig S1. Effects on food intake, body weight and survival by macronutrient free diets and effects on kidney function upon subsequent renal ischemia reperfusion injury.**
- **Fig S2. PCA of all probes on the microarray chip.**
- **Fig S3. Schematic overview of the experimental design.**
- **Fig S4. PCR data of mRNA expression levels of genes related to significantly regulated transcription factors.**
- **Fig S5. Representative Western blots of kidney extracts for both phosphorylated (A) and total (B) ribosomal protein S6 with β-actin as a loading control used for the relative quantification in Figure 4.**
- **Table S1. List of genes corresponding to the DEPS found in common between all dietary interventions combined.**
- **Table S2. Top 10 overrepresented canonical pathways after fasting, dietary restriction and macronutrient free diets individually ranked by their –log P-value.**
- **Table S3. Top 10 overrepresented canonical pathways after the meta-analysis including fasting, dietary restriction and macronutrient free diets ranked by their –log P-value.**
- **Table S4**. **Composition and energy content of the individual diets.**
- **Table S5. Overview of the dietary interventions, the groups and numbers of mice used for phenotypical and transcriptional endpoints.**

**Figure S1.**

**
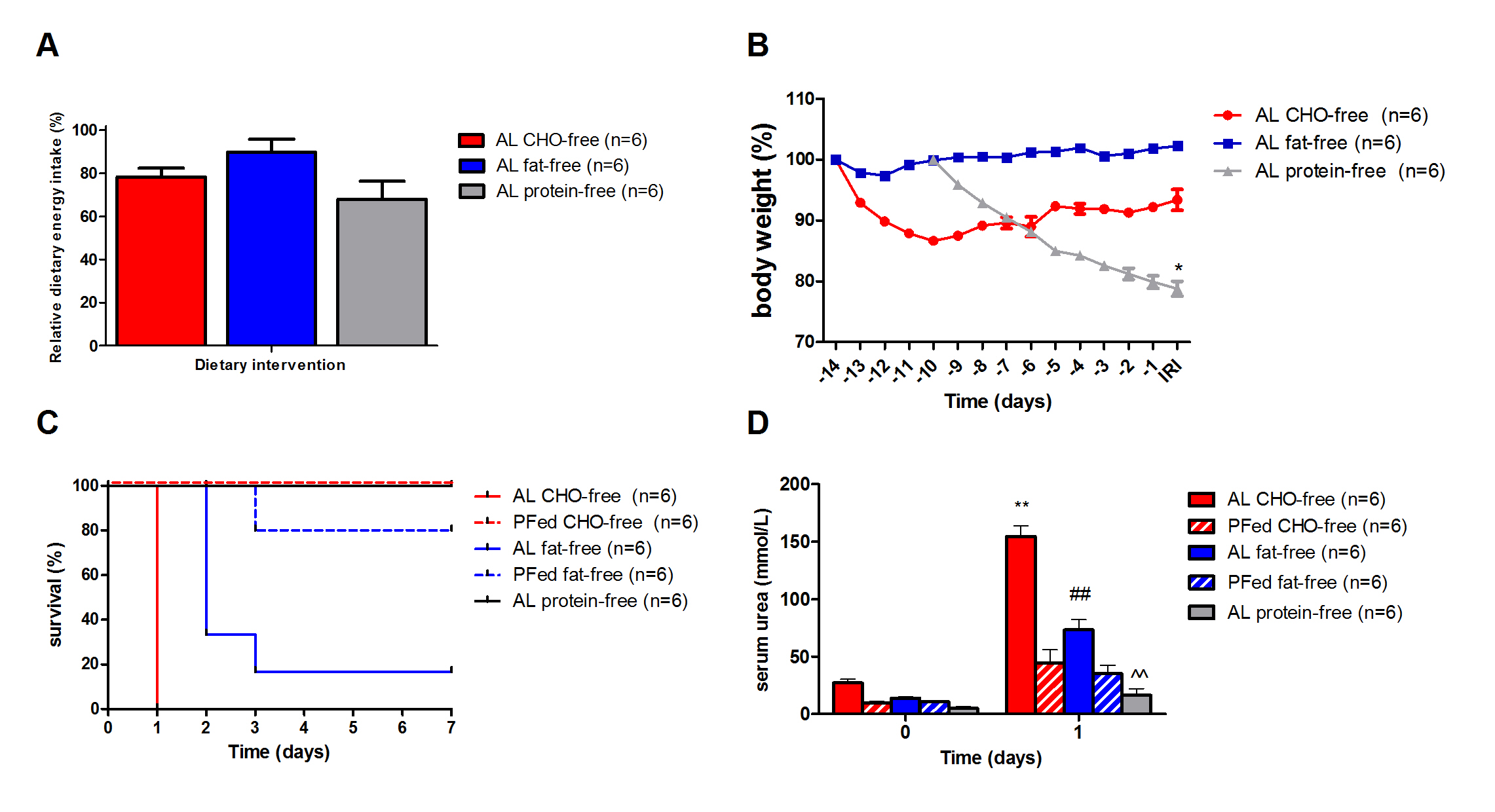
**

**Fig S1. Effects on food intake, body weight and survival by macronutrient free diets and effects on kidney function upon subsequent renal ischemia reperfusion injury.** **(A)** Relative to their control food intake during the 7 day acclimatization period, mice consumed (cumulatively during the whole intervention period) 21.7% less of the CHO-free diet, 10.1% of the fat-free diet and 32.7% of the protein-free diet. **(B)** Body weight of mice fed a CHO-free diet decreased during the 14-day period with 6.7%, while mice on a 14-day fat-free diet showed an increase in body weight with 2.3%. Mice fed a protein-free diet for 10 days lost more than 20% of their body weight on day 10. Because of this substantial loss of body weight, the protein-free diet was limited to a period of 10 days or less in the subsequent ischemia-reperfusion experiments. **(C)** Mouse mortality rates upon renal ischemia-reperfusion injury after both the CHO- and fat-free diet were significantly higher than their PFed controls (P<0.05). Mice fed a protein-free diet for 10 days showed a 100% survival following renal IRI. **(D)** Kidney function as determined by serum urea levels on day 1 after induction of renal IRI were significantly worsened in both the CHO-free (P<0.05) and fat-free (P<0.05) groups compared to PFed groups. Mice fed a protein-free diet showed lower serum urea levels one day after induction of IRI compared with CHO-free (P<0.01), the PFed CHO-free (P<0.05), the fat-free (P<0.01) and the PFed fat-free group (P<0.05). ** = P<0.05 compared to AL CHO-free diet on day 0. ## = P<0.05 compared to AL fat-free diet on day 0. ^^ = P<0.05 compared to AL protein-free diet on day 0.

**Figure S2**.


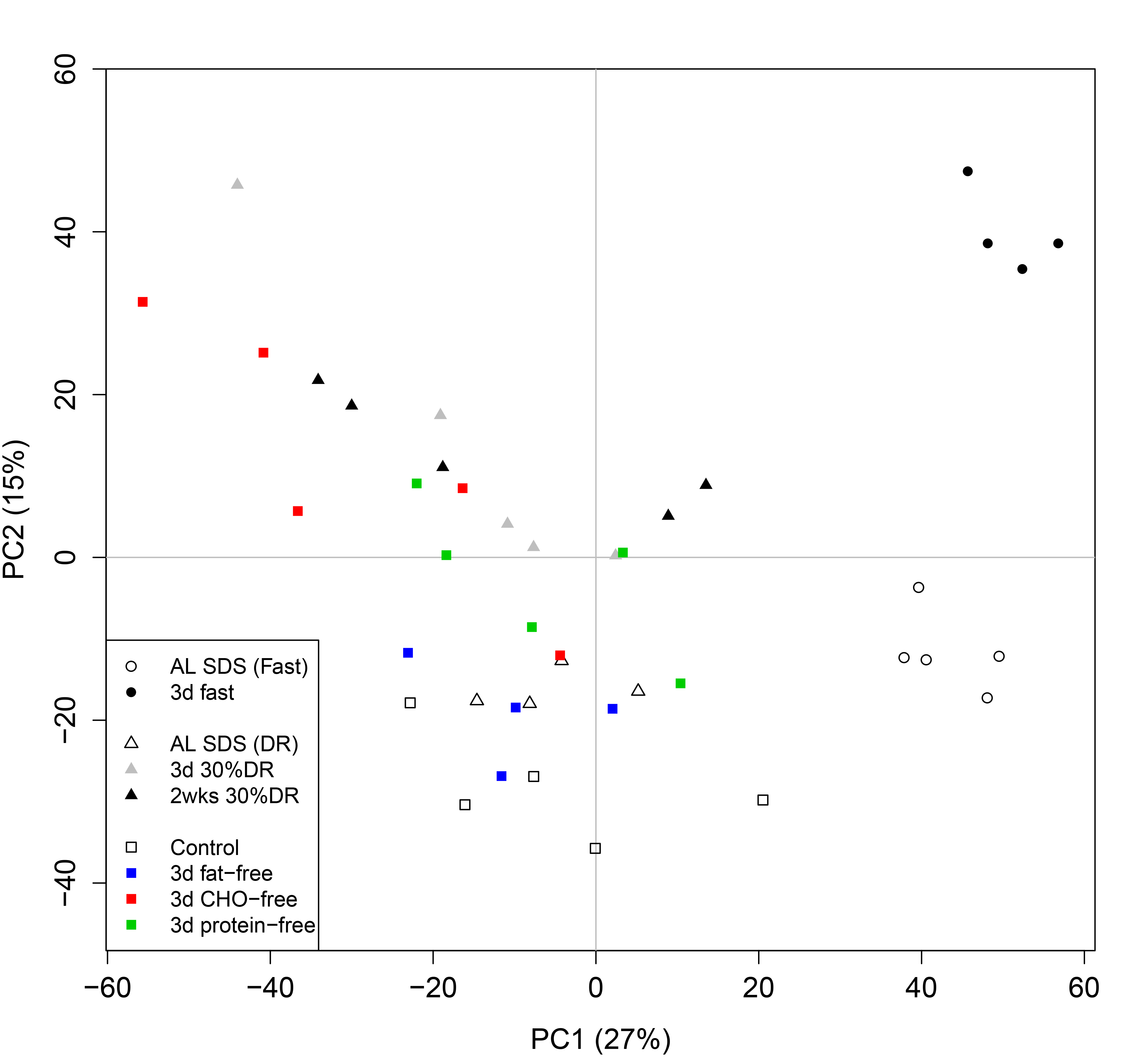


**Fig S2. PCA of all probes on the microarray chip.** This unbiased analysis shows the argest source of variability, PC1 is related to the date of hybridization. The 3-day fasting group and their corresponding control group were hybridized on a different date than the other groups. This result made the joint analysis of all datasets impracticable. PC2 explains only 15% of the variability, and it is related to the dietary interventions compared to the control samples. AL SDS (Fast)= control group of the 3-day fasting mice. AL SDS (DR) = control group of the 2 weeks 30%DR mice. Control = control group of the macronutrient-free diets.

**Figure S3.**


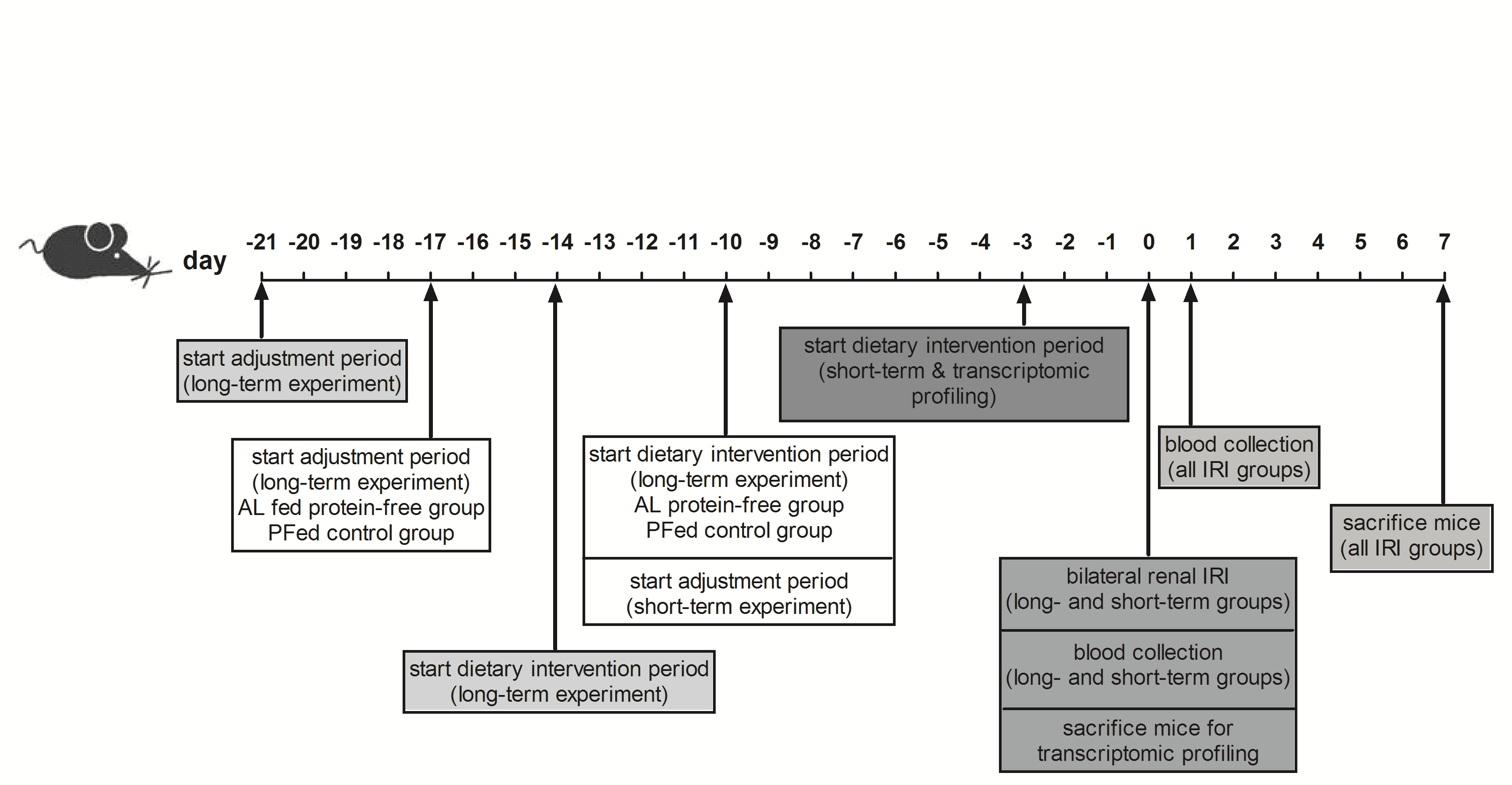


**Fig S3. Schematic overview of the experimental design.** After an acclimation period for all groups of 7 days on SDS chow, the long-term experiments started with diets given for either 10- or 14 days. At day -3 relative to induction of ischemia-reperfusion injury (IRI) the short-term dietary intervention and gene expression profiling experiments were started. At day 0, animals used for transcriptomic profiling were sacrificed for further analyses. In the other groups, blood collection was followed by induction of bilateral renal IRI for 37 minutes and collection of blood one day later. Mice were followed until day 7 after IRI, after which they were sacrificed.

**Figure S4.**

**
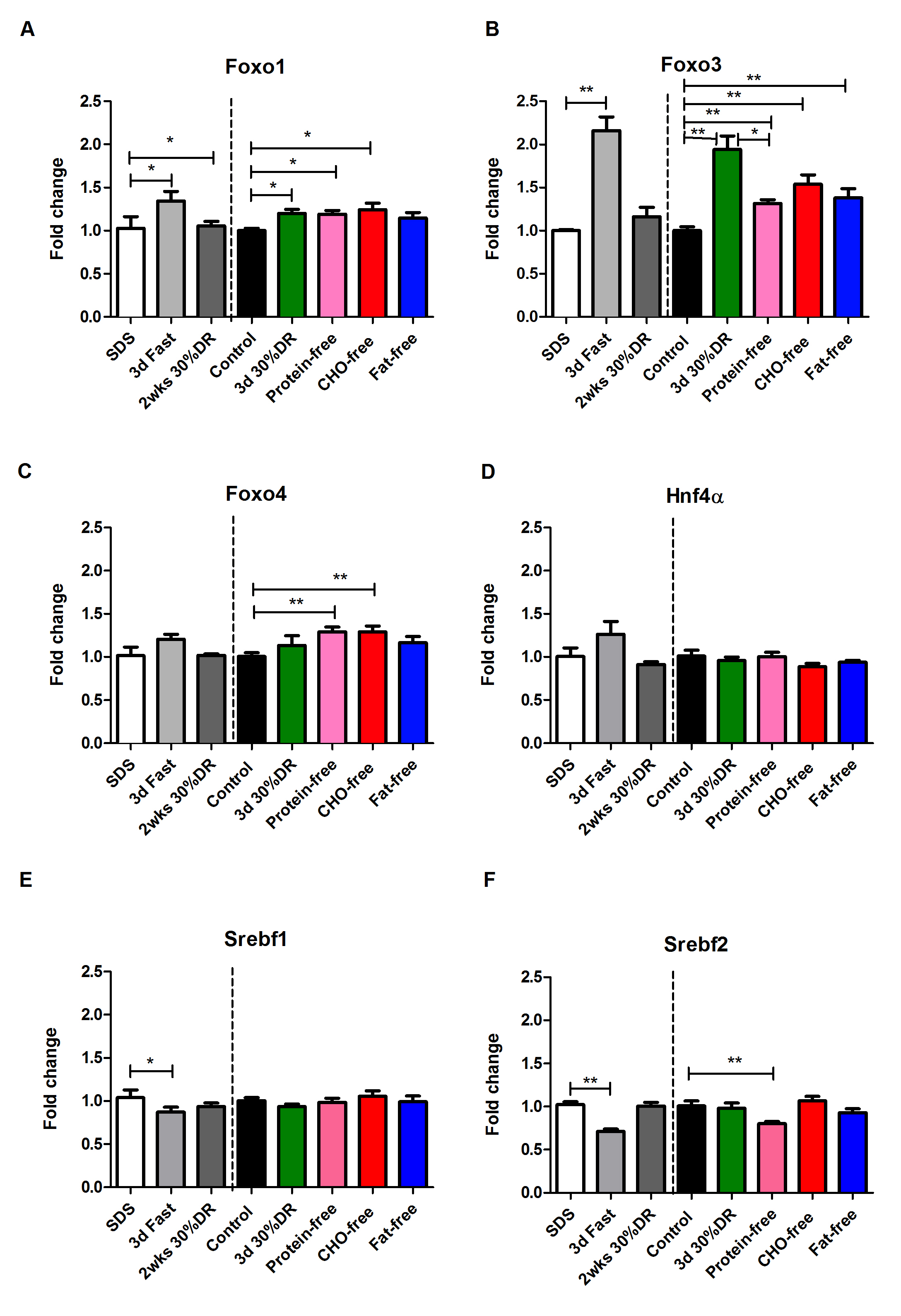
**

**Fig S4. PCR data of mRNA expression levels of genes related to significantly regulated transcription factors. (A)** FOXO1 was significantly higher after all diets except the fat-free diet. **(B)** FOXO3 showed significant upregulation after all diets except the 2 weeks 30% DR. **(C)** FOXO4 expression levels were significant higher after 3 days of 30% DR and 3-days of a CHO-free diet. **(D)** Hnf4α was not significantly regulated after any of the dietary interventions. **(E)** Srebf1 was down-regulated after 3 days of fasting, while **(F)** Srebf2 was significantly down-regulated after both 3 days of fasting and a 3-day protein-free diet. FOXO = forkhead box O; Hnf4α = hepatocyte nuclear factor 4-alpha; Srebf = sterol regulatory element-binding transcription factor 1. * = P<0.05; ** = P<0.01.

**Figure S5**

**A** **B**

| Slot | Dietary intervention |
| --- | --- |
| Mr | Marker lane |
| 1 | Control |
| 2 | CHO-free |
| 3 | Fat-free |
| 4 | Protein-free |
| 5 | 3-day 30% DR |
| 6 | 2-week 30% DR |
| 7 | SDS control |
| 8 | 3-day fasting |
| 9 | Reference sample |

**Fig S5**. **Representative Western blots** **of kidney extracts for both phosphorylated (A) and total (B) ribosomal protein S6 with** β**-actin as a loading control used for the relative quantification in Figure 4.** The depicted exemplary blots show a full set of kidney extracts from each indicated diet group (lanes *1 – 8*) next to a marker lane (lane *Mr*) and a reference sample (lane *9*). In total, four such sets of Western blots were generated, each with four unique complete series for each diet group. For interblot comparison the exact same amount of the reference sample (lane 9) was loaded on each blot. The relative phosphorylation ratios depicted in Figure 4. are derived from the quantified signal of the main band in each lane, subtracted with background and corrected for both the sample dependent β-actin signal and the blot dependent signal of the reference sample.

**Table S1. List of genes corresponding to the DEPS found in common between all dietary interventions combined.**

| **Affymetrix ID** | **Entrez Gene ID** | **Symbol** | **Gene Name** | **Log Ratio** |
| --- | --- | --- | --- | --- |
| 1415802_PM_at | 20501 | SLC16A1 | solute carrier family 16 (monocarboxylate transporter), member 1 | -0,488 |
| 1415828_PM_a_at | 28146 | SERP1 | stress-associated endoplasmic reticulum protein 1 | -0,639 |
| 1416414_PM_at | 100952 | EMILIN1 | elastin microfibril interfacer 1 | -0,623 |
| 1416630_PM_at | 15903 | ID3 | inhibitor of DNA binding 3, dominant negative helix-loop-helix protein | 1,008 |
| 1417150_PM_at | 15567 | SLC6A4 | solute carrier family 6 (neurotransmitter transporter), member 4 | 0,537 |
| 1418591_PM_at | 58233 | DNAJA4 | DnaJ (Hsp40) homolog, subfamily A, member 4 | -0,709 |
| 1419040_PM_at | 56448 | Cyp2d22 | cytochrome P450, family 2, subfamily d, polypeptide 22 | 1,033 |
| 1422974_PM_at | 23959 | NT5E | 5'-nucleotidase, ecto (CD73) | -1,037 |
| 1423392_PM_at | 29876 | CLIC4 | chloride intracellular channel 4 | -0,782 |
| 1423627_PM_at | 18104 | NQO1 | NAD(P)H dehydrogenase, quinone 1 | 1,024 |
| 1424618_PM_at | 15445 | HPD | 4-hydroxyphenylpyruvate dioxygenase | 1,064 |
| 1424983_PM_a_at | 78832 | CACUL1 | CDK2-associated, cullin domain 1 | -0,746 |
| 1426568_PM_at | 117591 | SLC2A9 | solute carrier family 2 (facilitated glucose transporter), member 9 | 0,684 |
| 1426896_PM_at | 59057 | ZNF24 | zinc finger protein 24 | -0,495 |
| 1427364_PM_a_at | 18263 | ODC1 | ornithine decarboxylase 1 | -0,975 |
| 1427931_PM_s_at | 216134 | PDXK | pyridoxal (pyridoxine, vitamin B6) kinase | 1,251 |
| 1428332_PM_at | 216505 | PIK3IP1 | phosphoinositide-3-kinase interacting protein 1 | 0,828 |
| 1433557_PM_at | 52609 | CBX7 | chromobox homolog 7 | 0,618 |
| 1434437_PM_x_at | 20135 | RRM2 | ribonucleotide reductase M2 | -0,128 |
| 1434974_PM_at | 228026 | PDK1 | pyruvate dehydrogenase kinase, isozyme 1 | 0,683 |
| 1436893_PM_a_at | 57438 | MARCH7 | membrane-associated ring finger (C3HC4) 7, E3 ubiquitin protein ligase | 0,585 |
| 1438211_PM_s_at | 13170 | DBP | D site of albumin promoter (albumin D-box) binding protein | 1,768 |
| 1439797_PM_at | 19015 | PPARD | peroxisome proliferator-activated receptor delta | -0,838 |
| 1443870_PM_at | 239273 | ABCC4 | ATP-binding cassette, sub-family C (CFTR/MRP), member 4 | 1,063 |
| 1449183_PM_at | 12846 | COMT | catechol-O-methyltransferase | -0,944 |
| 1449460_PM_at | 142688 | ASB13 | ankyrin repeat and SOCS box containing 13 | -0,686 |
| 1449848_PM_at | 14675 | GNA14 | guanine nucleotide binding protein (G protein), alpha 14 | -0,832 |
| 1451122_PM_at | 319554 | IDI1 | isopentenyl-diphosphate delta isomerase 1 | -1,173 |
| 1452264_PM_at | 209039 | TNS2 | tensin 2 | 0,757 |
| 1454709_PM_at | 100201 | TMEM64 | transmembrane protein 64 | -0,867 |
| 1455293_PM_at | 235497 | LEO1 | LEO1 homolog, Paf1/RNA polymerase II complex component | -0,727 |
| 1455343_PM_at | 233765 | PLEKHA7 | pleckstrin homology domain containing, family A member 7 | 0,922 |
| 1455393_PM_at | 12870 | CP | ceruloplasmin (ferroxidase) | 1,037 |
| 1455490_PM_at | 18703 | PIGR | polymeric immunoglobulin receptor | 0,924 |
| 1457689_PM_at | 319934 | SBF2 | SET binding factor 2 | 0,761 |
| 1458176_PM_at | 18628 | PER3 | period circadian clock 3 | 1,072 |
| 1460239_PM_at | 66109 | TSPAN13 | tetraspanin 13 | -0,925 |

The Afymmetrix probe sets, their corresponding Entrez Gene ID, symbol, gene name and log ratio are depicted in ascending order of the Affymetrix probe set. Only the probe sets with a corresponding gene ID are listed, therefore probe sets corresponding to the same gene ID are excluded. Log Ratio = log transformed fold change.

**Table S2. Top 10 overrepresented canonical pathways after fasting, dietary restriction and macronutrient free diets individually ranked by their –log P-value.**

| ***3-day FASTING*** |  | | |  | |
| --- | --- | --- | --- | --- | --- |
| **Canonical Pathway** | **P-value** | | | **Genes Ratio** | |
| Superpathway Cholesterol Biosynthesis | 3.41E-08 | | | 14/87 (16.1%) | |
| Cholesterol Biosynthesis I / II / III | 2.18E-06 | | | 8/40 (20.0%) | |
| LXR / RXR Activation | 2.20E-06 | | | 27/139 (19.4%) | |
| NRF2-mediated Oxidative Stress Response | 6.27E-06 | | | 34/195 (17.4%) | |
| LPS/IL-1 Mediated Inhibition of RXR Function | 7.46E-06 | | | 39/245 (15.9%) | |
| Acute Phase Response Signaling | 7.24E-05 | | | 30/181 (16.6%) | |
| GADD45 Signaling | 1.25E-04 | | | 8/24 (33.3%) | |
| AMPK Signaling | 1.25E-04 | | | 25/180 (13.9%) | |
| VDR/RXR Activation | 2.18E-04 | | | 17/88 (19.3%) | |
| Xenobiotic Metabolism Signaling | 3.00E-04 | | | 42/304 (13.8%) | |
| ***2-week 30% DR*** |  | | |  | |
| **Canonical Pathway** | **P-value** | | | **Genes Ratio** | |
| Circadian Rhythm Signaling | 1.41E-05 | | | 6/38 (15.8%) | |
| Aldosterone Signaling Epithelial Cells | 3.20E-05 | | | 11/168 (6.5%) | |
| Guanosine Nucleotides Degradation II | 7.85E-04 | | | 3/22 (13.6%) | |
| Urate Biosynthesis | 1.01E-03 | | | 3/22 (13.6%) | |
| Phenylalanine Degradation IV | 1.27E-03 | | | 3/39 (7.7%) | |
| 2-ketoglutarate Dehydrogenase Complex | 1.48E-03 | | | 2/9 (22.2%) | |
| Adenosine Nucleotides Degradation II | 1.57E-03 | | | 3/26 (11.5%) | |
| NRF2-mediated Oxidative Stress Response | 2.39E-03 | | | 9/195 (4.6%) | |
| Protein Ubiquitination Pathway | 2.63E-03 | | | 11/270 (4.1%) | |
| Purine Nucleotides Degradation II | 2.71E-03 | | | 3/35 (8.6%) | |
| ***3-day PROTEIN-FREE*** | |  | |  | |
| **Canonical Pathway** | | **P-value** | | **Genes Ratio** | |
| Intrinsic Prothrombin Activation Pathway | | 5.18E-04 | | 4/37 (10.8%) | |
| Superpathway Cholesterol Biosynthesis | | 6.79E-04 | | 4/87 (4.6%) | |
| LPS/IL-1 Mediated Inhibition of RXR Function | | 8.81E-04 | | 10/245 (4.1%) | |
| Mevalonate Pathway I | | 9.87E-04 | | 3/29 (10.3%) | |
| Creatine-phosphate Biosynthesis | | 1.77E-03 | | 2/9 (22.2%) | |
| Superpathway of Geranylgeranyl | | 2.02E-03 | | 3/37 (8.1%) | |
| PXR/RXR Activation | | 2.12E-03 | | 5/92 (5.4%) | |
| GADD45 Signaling | | 2.35E-03 | | 3/24 (12.5%) | |
| Tryptophan Degradation | | 4.84E-03 | | 2/18 (11.1%) | |
| Nicotine Degradation II | | 9.33E-03 | | 4/85 (4.7%) | |
| ***3-day CARBOHYDRATE-FREE*** | | |  | |  |
| **Canonical Pathway** | | | **P-value** | | **Genes Ratio** |
| Histamine Degradation | | | 9.52E-05 | | 6/29 (20.7%) |
| Pyrimidine Deoxygenase De Novo Biosynthesis I | | | 1.57E-04 | | 6/34 (17.6%) |
| Superpathway of Serine and Glycine Biosynthesis I | | | 2.57E-04 | | 4/18 (22.2%) |
| Glycolysis I | | | 6.98E-04 | | 7/41 (17.1%) |
| NRF2-mediated Oxidative Stress Response | | | 8.17E-04 | | 24/195 (12.3%) |
| Cholesterol Biosynthesis I / II / III | | | 1.04E-03 | | 5/40 (12.5%) |
| Colanic Acid Building Blocks Biosynthesis | | | 1.52E-03 | | 5/36 (13.9%) |
| Fatty Acid β-oxidation | | | 1.52E-03 | | 5/21 (23.8%) |
| Protein Ubiquitination Pathway | | | 1.58E-03 | | 30/270 (11.1%) |
| Folate Transformations I | | | 1.84E-03 | | 4/32 (12.5%) |
| ***3-day 30% DR*** | | |  | |  |
| **Canonical Pathway** | | | **P-value** | | **Genes Ratio** |
| Dopamine Degradation | | | 1.08E-04 | | 5/35 (14.3%) |
| Aryl Hydrocarbon Receptor Signaling | | | 1.37E-04 | | 9/140 (6.4%) |
| LPS/IL-1 Mediated Inhibition of RXR Function | | | 2.48E-04 | | 11/221 (5.0%) |
| PXR/RXR Activation  Circadian Rhythm Signaling | | | 2.63E-04  1.03E-03 | | 6/65 (9.2%)  4/33 (12.1%) |
| NRF2-mediated Oxidative Stress Response | | | 1.42E-03 | | 9/193 (4.7%) |
| Tyrosine Degradation I | | | 1.82E-03 | | 2/5 (40.0%) |
| Histamine Degradation | | | 2.09E-03 | | 3/19 (15.8%) |
| Noradrenaline and Adrenaline Degradation | | | 2.14E-03 | | 4/40 (10.0%) |
| Adipogenesis Pathway | | | 2.52E-03 | | 7/134 (5.2%) |

Top 10 overrepresented pathways after dietary restriction and macronutrient free diets, with the exception of the 3-day fat-free diet that showed no differentially expressed probe sets. Analysis revealed no pathways regulated in common between the three protective diets. Furthermore, no pathways were oppositely regulated in the non-protective CHO-free diet. Genes ratio is the number and percentage of genes differentially expressed in ratio to the total number of genes involved in the pathway.

**Table S3. Top 10 overrepresented canonical pathways after the meta-analysis including fasting, dietary restriction and macronutrient free diets ranked by their –log P-value.**

| ***Meta-analysis II (including 3-day 30% DR)*** |  |  |  |  |
| --- | --- | --- | --- | --- |
| **Canonical Pathway** | **Pathway Category** | **P-value** | **Genes Ratio** | **Z-score** |
| LPS/IL-1 Mediated Inhibition of RXR Function | Nuclear Receptor Signaling | 4.68E-05 | 10/221 (4.5%) | N/A |
| NRF2-mediated Oxidative Stress Response | Cellular Stress and Injury | 8.92E-05 | 9/193 (4.7%) | N/A |
| PXR/RXR Activation | Nuclear Receptor Signaling | 3.59E-04 | 5/65 (7.7%) | N/A |
| Noradrenaline and Adrenaline Degradation | Degradation/Utilization/ Assimilation; Hormones | 5.25E-04 | 4.40 (10.0%) | N/A |
|  |  |  |  |  |
| Aryl Hydrocarbon Receptor Signaling | Cell Cycle Regulation; Apoptosis; Xenobiotic Metabolism, Nuclear Receptor Signaling | 2.08E-03 | 6/140 (4.3%) | N/A |
| Superpathway of Cholesterol Biosynthesis | Fatty Acids and Lipids Biosynthesis, Sterol Biosynthesis | 2.23E-03 | 3/28 (10.7%) | N/A |
| Glutathione-mediated Detoxification | Degradation/Utilization/ Assimilation; Detoxification | 2.47E-03 | 3/29 (10.3%) | N/A |
| Circadian Rhythm Signaling | Neurotransmitters and Other Nervous System Signaling | 3.59E-03 | 3/33 (9.1%) | N/A |
| Retinoate Biosynthesis I | Vitamin (A) Biosynthesis; Cofactors, Prosthetic Groups and Electron Carriers Biosynthesis | 3.91E-03 | 3/34 (8.8%) | N/A |
| Dopamine Degradation | Degradation/Utilization/ Assimilation; Amines and Polyamines Degradation | 4.25E-03 | 3/35 (8.6%) | N/A |

The top 10 overrepresented pathways derived from the 279 DEPS in common between 3-days of fasting, 2 weeks 30%DR, 3 days of a protein-free and 3 days of a 30%DR diet. These pathways are mostly involved in regulation of nuclear receptor signalling (5 out of 10), biosynthesis signalling (2 out of 10) and cellular stress and injury (2 out of 10).

**Table S4**. **Composition and energy content of the individual diets.**

| **Ingredient** | ***Control*** | ***SDS-CRMP*** | ***Carbohydrate-free*** | ***Fat-free*** | ***Protein-free*** |
| --- | --- | --- | --- | --- | --- |
| Crude protein* (%/g)  Casein, lactic (%/g) | 20.0 | 16.4 | 99.2 | 31.3 | 0 |
|  |  |  |  |  |  |
| **Total kcal/g** | **3.8** | **3.3** | **3.4** | **3.5** | **3.8** |
|  |  |  |  |  |  |
| Protein (g/kg) | 179.0 | 183.5 | 887.9 | 280.1 | 0.0 |
| Carbohydrate (g/kg) | 710.0 | 574.0 | 1.0 | 710.0 | 889.0 |
| Fat (g/kg) | 45.0 | 33.6 | 45.0 | 0.0 | 45.0 |
| Fiber (g/kg) | 50.0 | 24.8 | 50.0 | 50.0 | 50.0 |
|  |  |  |  |  |  |
| Protein (g%) | 17.0 | 22.0 | 77.2 | 24.9 | 0 |
| Carbohydrate (g%) | 67.3 | 68.9 | 0.1 | 63.1 | 86.2 |
| Fat (g%) | 4.3 | 9.1 | 3.9 | 0.0 | 4.4 |
| Fiber (g%) | 4.7 | 4.2 | 4.3 | 4.4 | 4.8 |
|  |  |  |  |  |  |
| Protein (kcal/kg) | 716 | 734 | 3551 | 1121 | 0 |
| Carbohydrate (kcal/kg) | 2840 | 2296 | 4 | 2840 | 3556 |
| Fat (kcal/kg) | 405 | 303 | 405 | 0 | 405 |
|  |  |  |  |  |  |
| Protein (kcal%) | 18 | 22 | 90 | 28 | 0 |
| Carbohydrate (kcal%) | 72 | 69 | 0 | 72 | 90 |
| Fat (kcal%) | 10 | 9 | 10 | 0 | 10 |

***protein sources: wheat, barley, soya, maize, potato protein**

**Table S5. Overview of the dietary interventions, the groups and numbers of mice used for phenotypical and transcriptional endpoints.**

(A) Overview IRI long-term experiments

| **Dietary Intervention** | **Model** | **Duration (days)** | **Number of mice** | **Follow-up (days)** | **Parameters measured** |
| --- | --- | --- | --- | --- | --- |
| Control SDS | C57BL/6 | 14 | 10 | 7 | Survival, body weight, kidney function |
| Control | C57BL/6 | 14 | 12 | 7 | Survival, body weight, kidney function |
| Fat-free | C57BL/6 | 14 | 6 | 7 | Survival, body weight, kidney function |
| Pair-fed fat-free | C57BL/6 | 14 | 6 | 7 | Survival, body weight, kidney function |
| Carbohydrate-free | C57BL/6 | 14 | 6 | 1 | Survival, body weight, kidney function |
| Pair-fed carbohydrate-free | C57BL/6 | 14 | 6 | 7 | Survival, body weight, kidney function |
| Protein-free | C57BL/6 | 10 | 6 | 7 | Survival, body weight, kidney function |

(B) Overview IRI short-term experiments

| **Dietary Intervention** | **Model** | **Duration (days)** | **Number of mice** | **Follow-up (days)** | **Parameters measured** |
| --- | --- | --- | --- | --- | --- |
| Control | C57BL/6 | 3 | 4 | 7 | Survival, body weight, kidney function |
| Protein-free | C57BL/6 | 3 | 4 | 7 | Survival, body weight, kidney function |
| AL Control (control to 3-day 30%DR) | C57BL/6 | 3 | 4 | 7 | Survival, body weight, kidney function |
| 3 days 30% DR | C57BL/6 | 3 | 6 | 7 | Survival, body weight, kidney function |

**(C) Overview of gene transcription experiments**

| **Dietary Intervention** | **Model** | **Duration (days)** | **Number of arrays** | **Follow-up (days)** | **Parameters measured** |
| --- | --- | --- | --- | --- | --- |
| Control SDS (control 2-week 30% DR) | C57BL/6 | 14 | 4 | 0 | Gene expression profiling in kidney tissue |
| 2-week 30% DR | C57BL/6 | 14 | 5 | 0 | Gene expression profiling in kidney tissue |
| Control SDS (control 3-day fasting) | C57BL/6 | 3 | 5 |  | Gene expression profiling in kidney tissue |
| 3-day fasting | C57BL/6 | 3 | 4 | 0 | Gene expression profiling in kidney tissue |
| Control | C57BL/6 | 3 | 5 | 0 | Gene expression profiling in kidney tissue |
| Protein-free | C57BL/6 | 3 | 5 | 0 | Gene expression profiling in kidney tissue |
| Fat-free | C57BL/6 | 3 | 4 | 0 | Gene expression profiling in kidney tissue |
| Carbohydrate-free | C57BL/6 | 3 | 5 | 0 | Gene expression profiling in kidney tissue |
